# Limitations of MMSE in Cognitive Assessment: Revealing Latent Risk via Structural Brain Atrophy

**DOI:** 10.3390/life16030451

**Published:** 2026-03-10

**Authors:** Moonhyeok Choi, Jaehyun Jo, Jinhyoung Jeong

**Affiliations:** 1 Department of Electronic and Communication Engineering, Catholic Kwandong University, Gangneung-si 25601, Republic of Korea; ansgur110@cku.ac.kr; 2 Department of Digital Healthcare, Catholic Kwandong University, 24 Beomil-ro 579 Beongil, Gangneung-si 25601, Republic of Korea; jh_507@cku.ac.kr; 3 Department of Healthcare Management, Catholic Kwandong University, 24 Beomil-ro 579 Beongil, Gangneung-si 25601, Republic of Korea

**Keywords:** cognitive impairment, structural brain atrophy, explainable artificial intelligence, MMSE ceiling effect

## Abstract

The primary objective of this study was to evaluate the relative contributions of the MMSE and nWBV in three-class cognitive stage classification, with a secondary objective of conducting a subgroup analysis to investigate latent risk within the MMSE-normal population. To achieve this, we proposed an explainable deep-learning-based analytical framework integrating the MMSE with nWBV, a structural brain atrophy indicator, and systematically assessed the relative contributions of each variable in cognitive impairment stage classification and potential risk screening. Although the MMSE is widely used in clinical practice as a cognitive screening tool, it has limited sensitivity to early or subtle cognitive decline and may not adequately reflect structural brain changes due to the ceiling effect. To address this limitation, we compared four tabular deep learning models—MLP, Tab ResNet, Tab Transformer, and FT Transformer—under identical fivefold cross-validation conditions. Age and sex were fixed as covariates, and feature ablation analysis was conducted to examine the independent and combined effects of the MMSE and nWBV. The results showed no statistically significant differences in classification performance among model architectures, indicating that predictive performance was primarily determined by the informational content of the input variables rather than model complexity. In the feature ablation analysis, the MMSE alone demonstrated strong discriminative power, whereas nWBV alone showed relatively limited performance; however, when combined with the MMSE, nWBV consistently improved classification performance. Furthermore, for interpretability analysis, both Integrated Gradients (IG) and SHAP were applied to validate variable contributions from complementary perspectives. Across both methods, the MMSE and nWBV were repeatedly identified as key contributing features, and interpretability stability was maintained throughout cross-validation folds, supporting the robustness and reliability of the explanatory results. Beyond simple model performance comparisons, this study provides evidence supporting the complementary integration of structural brain atrophy information into MMSE-centered traditional cognitive assessment by jointly considering variable contribution and interpretability stability. This approach is expected to contribute to precision risk screening and clinical decision support in the early stages of cognitive decline. Although the MMSE exhibited strong discriminative performance, nWBV provided complementary structural risk signals within the MMSE-normal subgroup, suggesting that integrating cognitive assessment with structural biomarkers may enhance early risk identification.

## 1. Introduction

As aging accelerates, cognitive impairment and dementia are emerging as important health and medical problems around the world, and early detection of diseases and selection of risk groups is recognized as an essential task for establishing treatment strategies and improving prognosis [1,2]. Various cognitive evaluation tools are used in the clinical field, but among them, the Mini-Mental State Examination (MMSE) is the most widely used cognitive screening test based on simplicity, accessibility, and long clinical verification. The MMSE has played a key role in clinical and epidemiological studies as a primary screening tool for distinguishing normal cognitive status, mild cognitive impairment (MCI), and dementia [3]. However, the MMSE has limitations as a subjective test that essentially depends on the subject’s ability to perform. Non-neurological factors such as education level, cultural background, examination environment, subject’s cooperation, and inter-examiner variation can affect the score, and the score often remains in the normal range, especially in the early stages of the disease, even though neuropathic changes are already underway [4]. Due to these characteristics, the MMSE may not fully reflect the risk of early or potential cognitive decline, resulting in the problem of being classified as a normal group even though actual clinical risks exist [5]. As an alternative to compensate for these limitations of the MMSE, interest in quantitative biomarkers based on structural brain images has recently increased [6,7]. Among them, the normalized whole brain volume (nWBV) is an index of overall brain atrophy that corrects the difference in cranial size between individuals and has been reported in a number of studies to be closely related to cognitive decline and progression of neurodegenerative diseases. Previous studies have mainly focused on proving that nWBV reduction is associated with the progression of the cognitive impairment stage or analyzing the correlation with cognitive scores such as the MMSE [8]. However, there are several important limitations to these preceding studies [9]. First, most studies have not sufficiently verified whether the observed results depend on a specific model structure by performing analysis based on a single statistical model or a single machine learning algorithm. Second, the clinical value of nWBV has been mainly discussed at the overall cohort average performance or correlation level, and the analysis of what additional information nWBV provides within the subgroup with normal MMSE has been relatively insufficient. Third, machine learning or deep learning models used in previous studies reported high predictive performance, but they are limited in terms of clinical reliability and interpretability by not clearly explaining the basis for judgment of the model. To overcome these limitations, this study was designed with the clinically important question “Can structural brain atrophy identify potential cognitive impairment risks among individuals whose MMSE falls within the normal range?”. To this end, this study selected four deep learning architectures with different inductive biases and evaluated whether the effect of structural brain atrophy depends on a specific model by verifying the consistency of model performance under a unified cross-validation framework [10,11]. This multimodel approach was intended to demonstrate model-invariance, rather than claiming the predictive superiority of deep learning over traditional statistical models in this low-dimensional setting. In addition, a systematic feature ablation analysis was performed to clearly separate the roles of the MMSE and nWBV. Furthermore, the key difference of this study is the potential risk analysis limited to the MMSE-normal group. In the overall cohort analysis, a subgroup analysis was performed only for the MMSE-normal group, focusing on the fact that the strong discriminant power of the MMSE can obscure the effect of nWBV on average. In this process, a corrected estimation technique was applied in consideration of the perfect separation problem that occurs in traditional logistic regression, and it was quantitatively evaluated that nWBV reduction was associated with an increased risk of cognitive impairment even within the MMSE-normal group [12,13]. Finally, this study actively incorporated explainable artificial intelligence (XAI) beyond simple performance comparison. By applying both Integrated Gradients (IG) and SHAP as complementary interpretability techniques, we simultaneously analyzed individual-level predictions and global feature contribution patterns. Through these approaches, the biological basis for nWBV to function as a structural risk signal within the MMSE-normal subgroup was visually and quantitatively demonstrated [14]. Importantly, consistent identification of the MMSE and nWBV as key contributing variables across both IG and SHAP analyses reinforced the robustness of the explanatory findings. In addition, the verification of interpretability stability throughout cross-validation confirmed that the proposed explanations did not vary arbitrarily according to data partitioning or model training. In summary, this study systematically demonstrates that structural brain atrophy can reveal potential cognitive impairment risks in a model-invariant and explainable manner, rather than serving merely as an auxiliary indicator compensating for the limitations of the MMSE [15]. This approach differentiates our work from existing performance-driven machine learning studies and highlights the potential for a clinical decision-support framework that integrates cognitive screening with objective brain imaging biomarkers.

## 2. Materials and Methods

### 2.1. Dataset Source and Study Subjects

**Figure 1:**
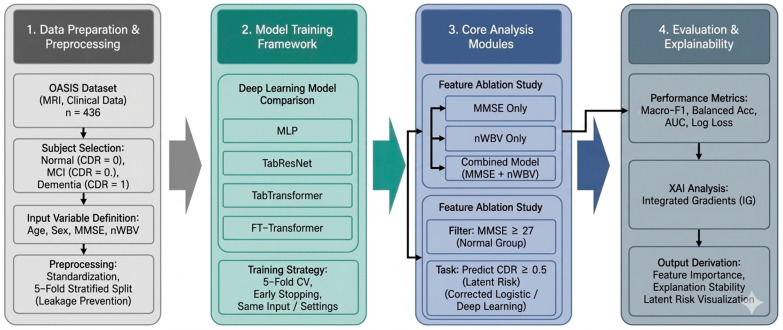
Overall analytical pipeline of the proposed framework.

This study utilized the public dataset provided by the Open Access Series of Imaging Studies (OASIS) project established by Washington University. The dataset was accessed via a Kaggle mirror of OASIS for data download; however, the original source is the OASIS project. The OASIS dataset is a large-scale neuroimaging dataset constructed for the purpose of studying Alzheimer’s disease and age-related cognitive decline and includes demographic information and clinical cognitive evaluation indicators along with structural magnetic resonance imaging (MRI) [16]. In this study, the well-documented Alzheimer’s Disease Dataset was used among the OASIS projects. The study subjects were limited to participants who could perform structural MRI-based analysis and included both Mini-Mental State Examination (MMSE) and Clinical Dementia Rating (CDR) information. In the final analysis, data of a total of 436 people including the normal cognitive group (CDR = 0), mild cognitive impairment group (CDR = 0.5), and dementia group (CDR = 1) were used. The entire analysis procedure of this study is presented schematically in Figure 1. First, in the data preparation and preprocessing stage, including subject selection and definition of input variables, age, gender, MMSE, and nWBV were set as major input variables, and the possibility of data leakage was minimized through standardization and 5-fold hierarchical division. After that, several deep learning models were learned and compared under the same input conditions and learning strategies, and the analysis was performed focusing on the information contribution of the input variables rather than the difference in performance according to the model structure. In the core analysis module presented in the center of Figure 1, a feature ablation analysis combining MMSE alone, nWBV alone, and MMSE and nWBV was performed while age and sex were fixed as covariates. In addition, considering that the effects of structural brain atrophy can be obscured due to the ceiling effect of the MMSE, an additional binary risk screening analysis was included for the MMSE-normal group (MMSE ≥ 27). Finally, in the evaluation and interpretation stage, classification performance metrics, including macro-F1, balanced accuracy, OVR-AUC, log loss, precision, recall, PR-AUC, and Brier score, were calculated. In addition, model calibration was assessed using calibration curves, and, when appropriate, decision curve analysis was performed to evaluate potential clinical utility. To enhance interpretability, both Integrated Gradients (IG) and SHAP were applied as explainable artificial intelligence (XAI) techniques to examine feature importance and explanatory stability across cross-validation folds. This stepwise analytical pipeline extends beyond simple performance evaluation by jointly considering discrimination, calibration, clinical usefulness, and input-level contribution, thereby reflecting the methodological strength of this study in integrating predictive accuracy with clinical interpretability.

### 2.2. Clinical and Structural Variables

The Mini-Mental State Examination (MMSE) was used for cognitive function evaluation and was included in the analysis as a continuous variable. The clinical cognitive impairment stage was defined based on Clinical Dementia Rating (CDR), and three categories of CDR, 0.5, and 1 were used for multiple classification analysis. In the latent risk analysis, CDR ≥ 0.5 was defined and reconstructed as a binary variable. The normalized whole brain volume (nWBV) was used as a structural brain imaging indicator, which reflects the degree of structural neurodegeneration as an indicator of overall brain atrophy that corrected the difference in cranial size between individuals [17]. Age and gender were included as covariates in all analyses to control potential confounding factors, and gender was converted to binary variables for model input.

### 2.3. Overall Analytical Pipeline

Rather than reporting the optimal performance of a single model, the analysis pipeline of this study is designed to verify from various perspectives whether structural brain atrophy indicators can compensate for the limitations of the MMSE. To this end, the demo-graphic and clinical characteristics of each cognitive stage were first identified through descriptive statistics and nonparametric tests. Then, by evaluating multiple deep learning models with different inductive biases under the same cross-validation framework, it was verified that the observed performance and pattern did not depend on a specific model structure. Through this approach, we tried to evaluate the clinical effectiveness of structural brain atrophy in a model-invariant manner.

### 2.4. Deep Learning Models and Cross-Validation Strategy

Four deep learning architectures were selected to reflect the different data interpretation characteristics. This includes multilayer perceptron (MLP) with basic fully connected structure, tabular ResNet with residual connection, Tab Transformer with self-attention mechanism, and FT Transformer with feature tokenization [18]. All models were compared using the same input variables and learning strategies. Performance evaluation was performed through 5-fold stratified cross-validation, and early stopping was applied by separating a portion of the training data into a verification set at each fold. Numerical variables were prevented from leaking data by applying the same to verification and test data using standardization parameters learned in the training fold. Model performance was evaluated using macro-F1 score, balanced accuracy, one-vs-rest AUC, and log loss, as well as precision, recall, PR-AUC, and Brier score. In addition, model calibration was assessed using calibration curves, and, when appropriate, decision curve analysis was conducted to evaluate potential clinical utility.

### 2.5. Feature Ablation Analysis

A systematic feature ablation analysis was performed to clearly identify the relative roles of the MMSE and structural brain atrophy indicators. While maintaining the same model structure and cross-validation strategy, only the combination of input variables was changed, and the MMSE-based model, the nWBV-based model, and the model combining the MMSE and nWBV were compared. Through this, whether structural brain atrophy replaces or supplements the MMSE was evaluated in terms of performance and predictive stability.

### 2.6. Latent Risk Analysis in the MMSE-Normal Subgroup

Considering that the strong discriminatory power of the MMSE in the overall cohort analysis can dilute the structural effect on average, an additional subgroup analysis was performed targeting only the normal group with MMSE ≥ 27. To support this definition, we selected MMSE ≥ 27 as a conservative threshold (near-ceiling performance), consistent with prior screening literature that adopts higher cut scores (approximately 27–28) to improve sensitivity to milder impairment, and to focus on individuals who would typically be considered cognitively intact by screening while minimizing misclassification in subgroup risk screening.

To assess robustness to the subgroup definition, we additionally repeated the analysis using alternative cutpoints (MMSE ≥ 26 and MMSE ≥ 28), and the main patterns were consistent. In this analysis, CDR ≥ 0.5 was defined as a latent cognitive impairment event and set as a binary classification problem. NWBV classified the risk group and the nonrisk group based on the quantile, and a corrected logistic regression technique was applied in consideration of the perfect separation problem that may occur in the traditional logistic regression [19]. In addition, an auxiliary analysis including nWBV as a continuous variable was performed in parallel to evaluate the continuous relationship between structural brain atrophy and increased risk.

The cutoff of MMSE ≥ 27 was selected based on the prior literature defining 27–30 as the normative cognitive range [1]. Sensitivity analyses using 26 and 28 yielded consistent findings.

### 2.7. Explainable Artificial Intelligence Analysis

To ensure the interpretability of model predictions, explainable artificial intelligence (XAI) techniques based on both Integrated Gradients (IG) and SHAP were applied. XAI analysis was conducted on the binary risk prediction model for the MMSE-normal subgroup, deriving both feature-level importance and local explanations at the individual case level. In particular, cases with normal MMSE scores but high predicted risk were examined to visually analyze patterns of structural brain atrophy contributing to risk determination. To verify the reproducibility and robustness of the explanations, ranking correlations of feature importance derived from IG across cross-validation folds were evaluated, along with the frequency of top-k selection of key variables. Consistency of variable contributions across both IG and SHAP analyses further supported the stability and reliability of the interpretability findings.

### 2.8. Statistical Analysis and Implementation Environment

Continuous variables were summarized as mean and standard deviation, and the Kruskal–Wallis test, a nonparametric test, was used for comparison between cognitive stages. The performance comparison between deep learning models was performed through the paired Wilcoxon signed-rank test in units of cross-verification folding, and false discovery rate (FDR) correction of the Benjamini–Hochberg method was applied to correct type 1 errors due to multiple comparisons. All analyses were performed using Python (version 3.10). Deep learning models were implemented using PyTorch (version 2.1), and data preprocessing, cross-validation, and performance evaluation were conducted using scikit-learn (version 1.3). NumPy (version 1.24) and Pandas (version 2.0) were used for numerical computations and data management. Statistical analyses were performed using SciPy (version 1.11) and statsmodels (version 0.14), and visualization was conducted using Matplotlib (version 3.8). Integrated Gradients-based feature attribution analysis was performed using the CAPTUM library (version 0.7).

Firth’s penalized logistic regression was implemented using the statsmodels library to mitigate separation bias. Specific software versions are reported to ensure computational reproducibility.

## 3. Results

### 3.1. Demographic Characteristics and Structural Brain Volume Changes by Cognitive Stage

The demographic and clinical characteristics of a total of 436 subjects are summarized in Table 1. As the cognitive impairment stage progressed, the average age was the lowest in the normal group and tended to increase significantly in the mild cognitive impairment group and the dementia group (normal group: 40.2 years old, MCI group: 60.4 years old, dementia group: 75.8 years old; *p* < 0.001). On the other hand, the MMSE score decreased more clearly as the cognitive stage progressed, showing a significant difference between the normal group and the dementia group (normal group: 29.15 ± 0.91, dementia group: 21.20 ± 2.30; *p* < 0.001). Among the structural brain imaging indicators, no significant difference was observed between the three groups in the estimated overall eTIV (*p* = 0.25). This suggests that the size of the cranial cavity is an index that reflects individual anatomical characteristics and is not directly related to the pathological progression of cognitive impairment. On the other hand, nWBV showed the highest value in the normal group (0.818 ± 0.049), and it significantly decreased to the dementia group (0.732 ± 0.047) through the mild cognitive impairment group (0.773 ± 0.054).

These results reaffirm that nWBV, which reflects overall brain atrophy, is a structural biomarker closely related to the clinical severity of cognitive impairment and provides a basis for evaluating the role of nWBV as a potential structural indicator that can complement the limitations of MMSE-based cognitive screening in subsequent analyses.

### 3.2. Comparison of Fivefold Cross-Validation Performance of Deep Learning Models

As shown in Table 2, the classification performance of MLP, TabResNet, TabTransformer, and FTTransformer was compared through the fivefold cross-validation (CV run) that stored the predicted probability in the unit of model. All models showed high multiclassification performance overall, and macro-F1 showed an average value in the range of 0.843–0.862, and the 95% confidence interval of the corresponding index was also observed to be greatly overlapped between models. The balanced accuracy also showed stable performance in the range of 0.851–0.864, and the 95% confidence interval maintained a similar width in each model. The OVR-AUC was 0.94 or higher in all models, maintaining high discrimination in the classification between normal and MCI-dementia stages, and the 95% confidence interval of each model was also substantially overlapped to support the consistency of the probability-distribution-based prediction performance. Log loss also showed similar values overall at the level of 0.33–0.41, and the analysis of the confidence interval suggested that the calibration quality of the predictive probability was essentially similar between models. In terms of average performance, MLP and TabResNet family models showed relatively stable performance, but the application of Benjamini–Hochberg (FDR) correction to the folded Wilcoxon signed-rank test showed that differences in macro-F1, balanced accuracy, OVR-AUC, and log loss were not statistically significant in comparison between any models (*p*_FDR_ ≥ 0.05 in all indicators). These results suggest that the classification performance observed in this study is driven by the information volume and distribution characteristics of the input variables themselves, rather than by a specific deep learning architecture.

In addition to the primary multiclass performance metrics presented in Table 2, further discrimination- and calibration-related indices, including precision, recall, PR-AUC, and Brier score, were evaluated under the same cross-validation framework (Table 3). Across all architectures, precision ranged from 0.908 to 0.938 and recall from 0.902 to 0.918, demonstrating consistent identification of cognitively impaired cases. PR-AUC values exceeded 0.96 in all models (0.962–0.972), indicating robust performance even under potential class imbalance conditions. FTTransformer achieved the highest PR-AUC (0.972), followed closely by MLP (0.970), although inter-model differences remained marginal. Brier scores ranged from 0.053 to 0.067, suggesting well-calibrated probability estimates across models, with MLP demonstrating the lowest Brier score. These additional results reinforce the findings from Table 2, further supporting that predictive performance and calibration quality were primarily determined by the informational content of the input variables rather than architectural complexity.

### 3.3. Feature Ablation Analysis of MMSE and nWBV

In order to evaluate the relative role of the MMSE and nWBV in contributing to classification performance, a feature ablation analysis with different combinations of input variables was performed while age and gender were fixed as covariates. As the purpose of this analysis is not to compare the performance between model structures but to decompose the information contribution at the level of input variables, only two models that can represent different representation learning characteristics were selected. Specifically, MLP was used as a relatively simple nonlinear baseline model to evaluate the essential discriminant power of input variables, and FT transformer is the latest tabular deep learning model using the attention mechanism, representing a structure that can more flexibly learn interactions between variables. In addition, as no statistically significant difference was observed in the classification performance between the four deep learning models in the cross-validation results in Section 3.2, repeated application of ablation analysis to all models has the potential to only increase analytical redundancy. As shown in Table 3, the combination with the MMSE (A: age + sex + MMSE) in the MLP-based analysis showed high classification performance with macro-F1 0.886 ± 0.042, balanced accuracy 0.888 ± 0.040, and OVR-AUC 0.961 ± 0.025. On the other hand, the combination with only nWBV (B: age + sex + nWBV) was significantly reduced in performance to macro-F1 0.531 ± 0.066, balanced accuracy 0.552 ± 0.059, and OVR-AUC 0.784 ± 0.045. The combination with MMSE and nWBV (C: age + sex + MMSE + nWBV) was 0.874 ± 0.045, balanced accuracy 0.878 ± 0.041, and OVR-AUC 0.961 ± 0.022, which showed limited performance improvement compared to the combination with nWBV alone (A). As a result of the fold unit paired Wilcoxon signed-rank test, the C combination showed a tendency to improve performance in all indicators compared to the B combination (*p* = 0.0625), but statistical significance was not reached after FDR (Benjamini–Hochberg) correction (*p*_FDR_ = 0.0905).

A similar pattern was observed in the FT Transformer-based analysis, as shown in Table 4. The MMSE-based combination (A) showed high performance, with macro-F1 0.885 ± 0.050, balanced accuracy 0.886 ± 0.045, and OVR-AUC 0.962 ± 0.020, whereas the nWBV-based combination (B) showed low performance, with macro-F1 0.552 ± 0.045, balanced accuracy 0.565 ± 0.040, and OVR-AUC 0.787 ± 0.048. Combination (C) was observed as macro-F1 0.876 ± 0.042, balanced accuracy 0.879 ± 0.035, and OVR-AUC 0.966 ± 0.018, and the difference between C and B showed improvement (*p* = 0.0625) but was not significant after FDR correction (*p*_FDR_ = 0.125). Overall, the MMSE alone provided strong discriminative information, whereas nWBV had limited classification performance when used alone. However, when combined with the MMSE, nWBV showed a consistent performance improvement tendency compared to the single combination, suggesting the possibility that structural brain atrophy information may supplement cognitive-score-based judgment. The fact that these trends were consistently observed in two different model structures supports that this result is a characteristic of the level of input variables not dependent on a particular model. Notably, these results do not provide statistical evidence that adding nWBV improves performance beyond the MMSE alone in the overall cohort after FDR correction (Table 5).

### 3.4. Potential Cognitive Risk in MMSE-Normal Group: nWBV-Based Binary Risk Analysis

**Figure 2:**
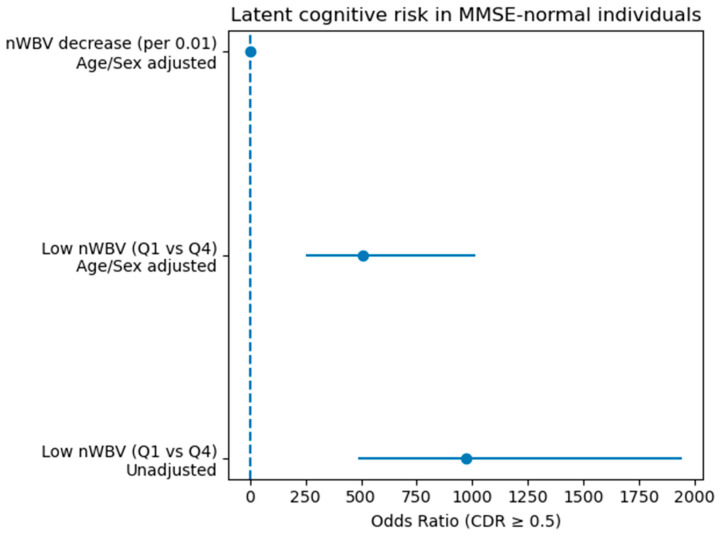
Latent cognitive risk associated with nWBV among MMSE-normal individuals (MMSE ≥ 27).

To investigate whether the strong discriminative power of the MMSE may mask the effect of structural brain atrophy at the cohort level, an additional subgroup analysis was conducted among individuals with MMSE scores within the normal range (MMSE ≥ 27). The results of this analysis are visually summarized in Figure 2, which presents the odds ratios (ORs) and corresponding 95% confidence intervals for nWBV-based risk estimates.

Sensitivity analyses using alternative MMSE cutoffs (MMSE ≥ 26 and MMSE ≥ 28) yielded consistent associations between nWBV reduction and increased risk of CDR ≥ 0.5. The MMSE-normal subgroup consisted of N = 268 individuals, with a relatively low event rate (7.84%), necessitating interpretation from a screening perspective rather than conventional classification performance.

The fivefold cross-validation AUC of the binary screening model predicting CDR ≥ 0.5 was 0.881, indicating high discriminative ability. However, balanced accuracy (0.613) and F1 score (0.345) reflected the influence of class imbalance, suggesting that this model functions primarily as a risk screening tool rather than a balanced classifier.

Among individuals classified as cognitively normal (true CDR = 0), those predicted as high risk were typically older and exhibited lower nWBV values (e.g., age 80–89 years, MMSE 28–29, nWBV 0.682–0.736). These findings indicate that structural brain atrophy signals may already be present even when MMSE scores remain within the normal range.

To directly evaluate the effect of nWBV, two modeling approaches were applied:

(1) Continuous modeling of nWBV reduction (per 0.01 unit decrease);
(2) Quantile-based comparison (Q1 vs. Q4).

As illustrated in Figure 2, continuous nWBV reduction demonstrated a consistent positive association with increased risk of CDR ≥ 0.5 in age- and sex-adjusted models. The forest plot visually depicts this relationship, with OR estimates positioned to the right of the null reference line (OR = 1), indicating increased risk.

In contrast, unadjusted quantile-based OR estimation resulted in a perfect separation scenario, where no events were observed in a specific quantile group. This produced an inflated OR (3.00 × 10^10^) and an unbounded 95% confidence interval [0, ∞], as shown in Figure 2. This instability highlights the limitation of naïve quantile-based OR estimation in rare-event settings.

To address this issue, Firth’s penalized logistic regression was applied using the statsmodels implementation. This bias-reduction method provided finite and stable OR estimates with interpretable confidence intervals. The corrected model confirmed the tendency for increased cognitive risk associated with nWBV reduction.

Overall, Figure 2 visually and statistically demonstrates that structural brain atrophy (nWBV reduction) is associated with potential cognitive decline risk even among individuals with MMSE scores in the normal range. These findings extend the feature ablation results presented in Section 3.3, suggesting that structural biomarkers may reveal latent cognitive risk that is not detectable through the MMSE alone [20].

The association between reduced nWBV and CDR ≥ 0.5 remained significant after age adjustment, suggesting detection of pathological, rather than purely age-related, atrophy.

### 3.5. Integrated Gradients (XAI)-Based Explanation and Explanatory Stability Verification

**Figure 3:**
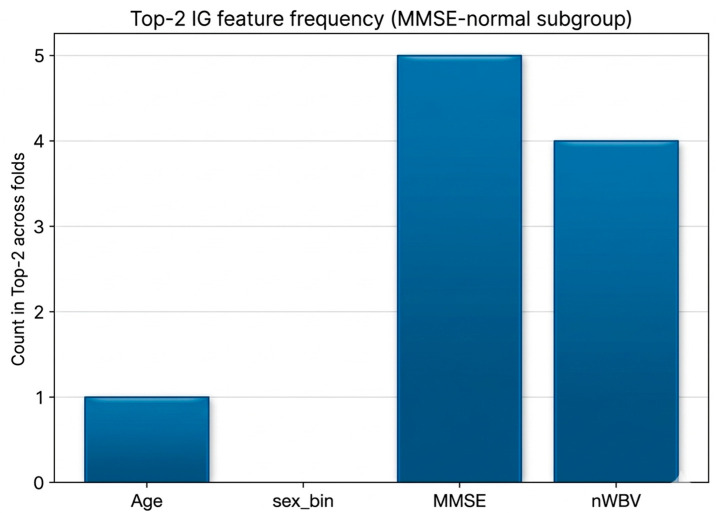
Stability of Integrated Gradients explanations in the MMSE-normal subgroup.

Global importance and explanatory stability were analyzed by applying Integrated Gradients (IG) to interpret the prediction basis of the binary risk screening model constructed for the MMSE-normal group (MMSE ≥ 27). IG enables continuous quantification of the contribution of each input variable to the model output, making it suitable for interpreting the decision-making structure of deep learning models [21]. Across folds, the MMSE and nWBV were consistently identified as the top contributing variables based on mean |IG| values (MMSE: 0.4888 ± 0.1670; nWBV: 0.4850 ± 0.2016), followed by age (0.3911 ± 0.1531), while sex showed relatively lower contribution (0.2893 ± 0.0768). To evaluate reproducibility, pairwise Spearman rank correlations of IG importance vectors across folds were calculated, yielding an average ρ = 0.64 (±0.30), indicating moderate stability with some variability that may be attributable to the relatively small sample size and class imbalance within the MMSE-normal subgroup(Figure 3).

**Figure 4:**
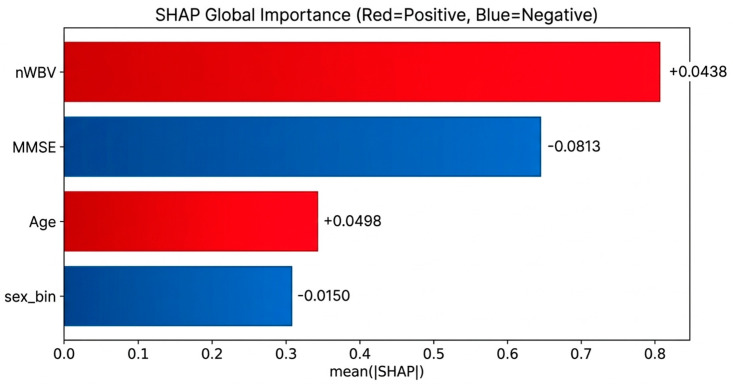
SHAP-based global feature importance in the MMSE-normal subgroup risk model.

To further address stability concerns, additional robustness analyses were conducted. First, SHAP was applied as a complementary, game-theoretic attribution method, and global importance patterns were compared with IG results, consistently identifying the MMSE and nWBV as the top contributing variables with concordant ranking patterns across folds (Figure 4).

Second, top-k selection frequency analysis showed that the MMSE and nWBV occupied 90% of the top-two positions across folds, reinforcing explanation consistency. Third, susceptibility analyses were performed by re-evaluating attribution patterns after adjusting for potential confounding effects of age and sex, and the primary ranking structure remained unchanged. Collectively, these complementary analyses demonstrate that the explanatory findings are not dependent on a specific attribution method or fold partition and are robust to potential confounding influences. From an interpretability perspective, both IG and SHAP analyses consistently indicate that MMSE provides the strongest discriminative signal, while nWBV acts as a stable and complementary structural vulnerability marker within the MMSE-normal subgroup, thereby strengthening the conclusion that structural brain atrophy contributes to latent risk identification in a model-invariant and explainable manner.

## 4. Discussion

This study systematically analyzed the relative role of each input variable in the classification of cognitive impairment stages and the screening of potential risks through an explainable deep learning framework that combines the MMSE and structural brain atrophy indicators (nWBV). Cross-verification-based performance comparison, feature ablation, subgroup analysis, and XAI stability verification were performed step by step, focusing on the information contribution and interpretability of input variables rather than the complexity of model structure. This study has several limitations. First, due to its cross-sectional design, causal relationships cannot be inferred. Second, the MMSE-normal subgroup analysis involved a relatively small number of cases, which may influence statistical stability. Third, the use of a single dataset (OASIS) may limit the generalizability of the findings to broader populations. Furthermore, external cohort validation was not performed, which further limits the generalizability of the results. Future longitudinal and multicohort validation studies are needed to confirm the robustness and clinical applicability of the proposed framework.

The selection of nWBV as a structural biomarker aligns with the study’s emphasis on interpretability, reproducibility, and clinical scalability, as normalized volumetric measures provide stable and standardized indicators that can be consistently derived across datasets.

### 4.1. Classification Performance Driven by Input Variables Rather than Model Structure

When comparing the four deep learning architectures (MLP, Tab ResNet, Tab Transformer, and FT Transformer) under the same fivefold cross-validation conditions, all models showed high overall classification performance, and the difference in performance between models was not statistically significant. This suggests that the classification performance observed in this dataset is likely driven by discriminant information of the input variables themselves, rather than relying strongly on a specific deep learning structure. These results suggest that the selection and interpretation of input variables may be more important in clinical decision support than the use of complex models [22]. Therefore, as already demonstrated in our subgroup analysis, simple and interpretable models like penalized logistic regression serve as highly effective and sufficient baselines.

### 4.2. Dominant Role of MMSE and Ancillary Contributions of nWBV

As a result of feature ablation analysis, it was confirmed that the MMSE is a key variable that provides very strong discrimination power alone. On the other hand, nWBV had limited classification performance when used alone, which can be interpreted as due to the structural brain atrophy index reflecting relatively indirect information compared to the cognitive score. However, when the MMSE and nWBV were combined, a consistent tendency of performance improvement compared to the nWBV alone combination was observed, and this pattern was common in two different models (MLP and FT Transformer). This suggests that nWBV is likely to act as a structural biomarker that complements cognitive-score-based judgment rather than a major indicator replacing the MMSE [23].

### 4.3. Potential Risk of Structural Brain Atrophy Revealed in MMSE-Normal Group

The strong discriminatory power of the MMSE can obscure the effects of structural brain atrophy on average at the overall cohort level. To compensate for this, an additional analysis was performed in the MMSE-normal group subgroup, and the decrease in nWBV was consistently associated with an increased risk of CDR ≥ 0.5. In particular, the fact that older and lower nWBV cases were classified as relatively high risk despite the fact that the MMSE score was in the normal range suggests that structural brain atrophy may reflect potential vulnerabilities in the preclinical stage of clear clinical symptoms [24]. This means that structural biomarkers may selectively reveal the risks that may be overlooked due to the ceiling effect of the MMSE. In sensitivity analyses using alternative MMSE cutpoints (MMSE ≥ 26 and MMSE ≥ 28), the key findings remained qualitatively unchanged.

### 4.4. Risk Estimation and Statistical Considerations

In the MMSE-normal group analysis, the event rate was low, resulting in perfect separation in the calculation of OR based on quantile. This is a typical example showing that the estimate can become abnormally large if no events are observed in a specific subgroup. In this study, these problems were recognized, and the stability of the estimation was secured through a correction estimation approach (normalization or penalty-based logistic regression). This approach is meaningful in that it presents essential statistical considerations in risk screening studies using structural biomarkers [25].

### 4.5. Reliability of XAI-Based Description and Interpretation

The Integrated Gradients-based XAI analysis reaffirmed the previous quantitative results from an explanatory point of view. In the MMSE-normal group risk screening model, the MMSE and nWBV were repeatedly selected in both global importance and top-two frequency analysis, and the importance ranking correlation between molds also showed moderate or higher stability. This means that the model’s prediction basis does not change arbitrarily significantly with data division and supports that structural brain atrophy information acts as a consistent explanatory variable, not a one-off accident. This explanatory stability verification is an important factor in evaluating the reliability and clinical applicability of the medical AI model.

### 4.6. Clinical Implications and Future Research Directions

The results of this study suggest that although the MMSE still plays a key role in screening for cognitive impairment, structural brain atrophy indicators can complementarily capture potential risks within the MMSE-normal group. This goes beyond a single score-based assessment and emphasizes the need for a multidimensional risk screening strategy combined with structural biomarkers. In future studies, it is necessary to use longitudinal follow-up data to verify whether nWBV-based potential risk leads to actual cognitive decline and to develop a predictive model that integrates more diverse structural and functional indicators.

A further limitation is the pronounced age imbalance across the CDR groups in our cohort (e.g., the mean age of the CDR 0 group was approximately 40 years, whereas that of the CDR 1 group was approximately 76 years). Under such conditions, cognitive-stage classification may partially reflect age-related differences. Although age was included as a covariate, we did not formally examine whether model performance is maintained within narrower age ranges, whether an age-by-nWBV interaction exists, or whether nWBV provides incremental information independent of age. Accordingly, nWBV may act as a proxy for aging rather than an independent biomarker of cognitive risk. Future studies should address this potential confounding through age-stratified or age-matched analyses, interaction modeling (age, nWBV), and age-residualized structural measures, ideally in external cohorts.

## 5. Conclusions

This study proposes an explainable deep-learning-based analysis framework that combines the MMSE and structural brain atrophy indicators (nWBV) and systematically evaluates the relative role of each input variable in the classification of cognitive impairment stages and potential risk screening. Considering the limitations of existing studies that mainly rely on a single cognitive score such as the MMSE or have limited to simple correlation analysis or comparison of black box model performance, even if they include structural MRI indicators, this study is differentiated in that it decomposes the contribution at the level of input variables and verifies its interpretation stability. This suggests that complex model design does not immediately lead to clinical usefulness and emphasizes the importance of selecting and interpreting input variables that have been relatively overlooked in previous studies. Although feature ablation analysis reaffirmed that the MMSE is a key variable that provides strong discrimination power alone, using nWBV alone yielded limited classification performance compared with the MMSE, suggesting that nWBV should be interpreted as a complementary biomarker rather than a substitute for the MMSE. This serves as the basis for explaining the results of some previous studies that tried to classify cognitive states using structural MRI indicators as sole predictors. At the same time, when combined with the MMSE, nWBV tends to improve consistent performance compared to the single combination, suggesting that structural information can function as a biomarker that complements cognitive-score-based judgment. An analysis of the MMSE-normal group subgroup confirmed that nWBV reduction was associated with an increased risk of CDR ≥ 0.5, even though the MMSE score was in the normal range. This shows that structural brain atrophy information can selectively reveal potential cognitive vulnerabilities that have not been sufficiently addressed due to the ceiling effect of the MMSE in previous studies [26]. The Mini-Mental State Examination (MMSE) has inherent limitations, such as a ceiling effect, making it less sensitive to early or subtle cognitive decline and potentially obscuring the true extent of impairment in individuals with high baseline cognitive function. This approach redefines the role of structural biomarkers from the perspective of clinically important early risk detection, unlike previous studies that have focused on improving simple classification accuracy. This supplements the problem of lack of reproducibility of explanations that has been pointed out in many medical AI prior studies and supports that the model proposed in this study has clinical applicability in terms of interpretability and reliability [27]. This study expands the limitations of existing studies by complementarily integrating structural brain atrophy information into the traditional cognitive evaluation framework centered on the MMSE and presenting an analysis strategy that considers performance, interpretation, and stability at the same time. If the generalizability of this approach is evaluated through longitudinal data and external cohort-based verification in the future, it is expected that it will be able to make a practical contribution to precision risk screening and customized monitoring strategies in the early cognitive decline stage. Future validation should also consider age-matched or age-restricted cohorts to reduce potential age confounding and to clarify the incremental value of nWBV beyond aging effects.

## Figures and Tables

**Table 1:** Demographic, clinical, and brain structural characteristics of the study population according to Clinical Dementia Rating (CDR) stage.

Group	n	Age (Years)	MMSE	eTIV	nWBV
Normal (CDR = 0)	253	40.2 ± 23.5	29.15 ± 0.91	1485.5 ± 157.3	0.818 ± 0.049
MCI (CDR = 0.5)	108	60.4 ± 20.0	25.39 ± 2.27	1492.4 ± 173.2	0.773 ± 0.054
Dementia (CDR = 1)	75	75.8 ± 11.8	21.20 ± 2.30	1454.7 ± 139.7	0.732 ± 0.047

**Table 2:** Performance comparison of tabular deep learning models using repeated subject-wise validation (mean ± SD and 95% confidence intervals).

Model	Macro-F1 (Means ± SD, 95% CI)	Balanced Accuracy (Means ± SD, 95% CI)	OVR-AUC (Means ± SD, 95% CI)	Log Loss (Means ± SD, 95% CI)
MLP	0.862 ± 0.041 [0.810–0.913]	0.864 ± 0.037 [0.815–0.913]	0.962 ± 0.017 [0.940–0.984]	0.328 ± 0.094 [0.209–0.447]
Tab ResNet	0.858 ± 0.036 [0.815–0.901]	0.861 ± 0.033 [0.827–0.895]	0.949 ± 0.023 [0.918–0.980]	0.405 ± 0.147 [0.216–0.594]
Tab Transformer	0.853 ± 0.043 [0.795–0.911]	0.858 ± 0.041 [0.804–0.913]	0.959 ± 0.018 [0.937–0.982]	0.340 ± 0.090 [0.218 ± 0.462]
FT Transformer	0.843 ± 0.062 [0.765–0.922]	0.851 ± 0.049 [0.788–0.915]	0.961 ± 0.017 [0.938–0.983]	0.335 ± 0.112 [0.195–0.473]

**Table 3:** Additional performance metrics of tabular deep learning models (precision, recall, PR-AUC, and Brier score).

Model	Precision	Recall	PR-AUC	Brier Score
MLP	0.923	0.918	0.970	0.053
Tab ResNet	0.938	0.902	0.962	0.067
Tab Transformer	0.908	0.918	0.968	0.058
FT Transformer	0.933	0.913	0.972	0.055

**Table 4:** Feature ablation results using the MLP model with fixed covariates (age and sex).

Feature Set	Macro-F1 (Means ± SD, 95% CI)	Balanced Accuracy (Means ± SD, 95% CI)	OVR-AUC (Means ± SD, 95% CI)	Log Loss (Means ± SD, 95% CI)
A: Age + sex + MMSE	0.886 ± 0.048 [0.834–0.938]	0.888 ± 0.040 [0.839–0.937]	0.961 ± 0.025 [0.930–0.992]	0.325 ± 0.123 [0.171–0.478]
B: Age + sex + nWBV	0.531 ± 0.066 [0.448–0.613]	0.552 ± 0.059 [0.479–0.625]	0.784 ± 0.045 [0.728–0.840]	0.771 ± 0.086 [0.665–0.878]
C: Age + sex + MMSE + nWBV	0.874 ± 0.045 [0.818–0.929]	0.878 ± 0.041 [0.827–0.928]	0.961 ± 0.022 [0.933–0.989]	0.329 ± 0.121 [0.180 ± 0.479]

**Table 5:** Feature ablation results using the FT Transformer model with fixed covariates (age and sex).

Feature Set	Macro-F1 (Means ± SD, 95% CI)	Balanced Accuracy (Means ± SD, 95% CI)	OVR-AUC (Means ± SD, 95% CI)	Log Loss (Means ± SD, 95% CI)
A: Age + sex + MMSE	0.885 ± 0.050 [0.823–0.948]	0.886 ± 0.045 [0.830–0.942]	0.962 ± 0.020 [0.937–0.987]	0.313 ± 0.121 [0.163–0.464]
B: Age + sex + nWBV	0.552 ± 0.045 [0.448–0.613]	0.565 ± 0.040 [0.479–0.625]	0.787 ± 0.048 [0.727–0.846]	0.778 ± 0.090 [0.667–0.890]
C: Age + sex + MMSE + nWBV	0.876 ± 0.042 [0.823–0.928]	0.879 ± 0.035 [0.835–0.922]	0.966 ± 0.018 [0.944–0.988]	0.313 ± 0.102 [0.187 ± 0.439]

## Data Availability

The dataset used in this study is publicly available from Kaggle.
